# Reflections on the design and application of ‘Surveypura’: a simulation-based pedagogical tool for quantitative research methods in public health and social sciences

**DOI:** 10.1186/s41077-023-00275-y

**Published:** 2024-01-07

**Authors:** Adithya Pradyumna, Mukta Gundi

**Affiliations:** Azim Premji University, Bengaluru, India

**Keywords:** Simulation, Quantitative research methods, Sampling, Data collection, Statistics, Pedagogy

## Abstract

It has been reported from various contexts that learning quantitative methods for public health and social research is challenging for students. Based on our observations of these challenges, we designed a simulation-based pedagogical tool called *Surveypura* to support classroom-based learning of quantitative research methods. The tool includes a large illustration of a fictional village with 155 houses, alongside data for each of the households. The features of the houses, household characteristics, and the village have been carefully designed to give the visual feel of an actual village and better assist the pedagogical process. The tool was used by five facilitators with their masters’ students at our university in courses on social research and epidemiology. Our observations of the sessions and interactions with facilitators and students suggested that the tool supported more engaged learning of quantitative research methods in a non-intimidating manner. We believe that *Surveypura* can be a useful simulation-based pedagogical tool to teach quantitative research methods in epidemiology and social sciences even in other contexts.

**Supplementary Information:**

The online version contains supplementary material available at 10.1186/s41077-023-00275-y.

## Introduction

Competency in quantitative methods is an important learning outcome in higher education in public health and the social sciences. Students are expected to demonstrate knowledge and skills towards conceptualizing a cross-sectional study, detailing the sampling plan, designing a survey questionnaire, collecting the data, analyzing it, and inferring from the quantitative results. They should be able to calculate rates (e.g., incidence of tuberculosis, prevalence of diabetes), ratios (e.g., maternal mortality ratio), proportions (e.g., proportion of respondents knowledgeable about malaria transmission), and measures of central tendency (e.g., average *z*-scores for height for a group of children), among other things. These skills are often taught as part of research methods courses in epidemiology or social sciences.

In these courses, students may learn the art and science of conducting a survey. While it requires the art of engaging with people, establishing cordial relationships with the community members, navigating the geography, and understanding the community’s needs and experiences in an ethically sensitive manner, it also requires a scientific temperament towards the study design, data collection and data analysis. These quantitative skills are usually taught in a classroom setting with limited scope for field-based learning.

Although concepts and skills such as simple random sampling, designing a structured questionnaire, calculating prevalence, and cross-tabulating variables may appear intuitive and straightforward to lecturers, our experience has shown that many students struggle with these topics. This is corroborated by the literature from other contexts. A US-based review found that social work students and faculty members “dreaded” statistics [1]. University students in the USA and Finland were found to have an adverse orientation towards quantitative methods, which was partially attributed to difficulties in learning quantitative methods, and this was irrespective of their subject and seniority [2]. In another study from Finland, students of education and social sciences rated quantitative methods as “harder” as compared to other subjects, and provided reasons including inadequate teaching, lack of application, the difficulty of subject matter, and adverse feelings to the subject [3]. A study by Kundu among school-going students in India has discussed the fear for mathematics among students, especially from socioeconomically marginalized backgrounds [4].

In courses on quantitative methods, facilitators often make pedagogical compromises by opting for online surveys and by using non-random samples due to constraints in time and access to communities. Additionally, datasets (in the form of spreadsheets) which are shared with students can be perceived as “detached” from their context—and some students may struggle to make sense of datasets. A study from the USA also showed that students preferred “real data” (for instance, data collected from students in the class) for learning as compared to an artificial dataset [5].

Our experience suggests that another challenge faced by educational institutions and their local area residents is the repeated involvement of local people for student exercises and projects—which can lead to community fatigue and can also have ethical implications. Based on all these observations and reflections, we felt that a different pedagogical approach is needed to help students better understand these important concepts within a classroom setting so that they can engage with cross-sectional studies and population data more confidently and effectively. This could also serve as preparation for field-based research exercises in the future.

Simulation as a pedagogical method of ‘action-learning’ has been tried and tested in a variety of domains in child and adult learning [6, 7]. It was reported that physical (tangible) and virtual simulators enhanced experiential, self-regulated learning and were cost-effective [7, 8]. The authors have been exposed to simulation games in community health and social sciences to understand complex challenges such as caste, power, poverty, and environmental determinants of health (the *Monsoon* game [9], and the *Chikkanahalli* game).

In order to augment the in-class action-learning experience for quantitative methods in social research, we developed a simulation pedagogical tool named “*Surveypura*” (italicized as “*Surveypura*”, when referring to the simulation pedagogical tool package, and not italicized as “Surveypura”, when referring to the fictional village of Surveypura). The idea of the tool is that the students would have to sample a few “households” from the village and “visit” them to collect data as described in the later sections to understand the process of conducting a cross-sectional study *Surveypura* being an in-class simulation tool, may provide a better opportunity for facilitators to closely observe and address conceptual concerns of students.

Our experience and a preliminary online search did not reveal any such available pedagogical tool for community-based quantitative research, which was an added motivator towards creating *Surveypura*. Also, we wanted to stay away from creating a virtual tool because we felt the need for physical interaction using props would enhance learning especially for programs in the social sciences. Finally, while this tool is not intended as a substitute for field-based learning, it can equip students with some essential conceptual understanding and skills before they conduct a field-based quantitative study.

## Designing the tool

### Step 1: broader considerations

The overall motivation was to provide a simulated experience of sampling some households from a population, visiting the households, collecting data, analyzing the data, and interpreting the findings. The aim was to design a tool that would help facilitate the learning of epidemiological and statistical concepts, and not necessarily the complexities of interacting with survey respondents in the field. The reasoning was that students should first gain conceptual clarity before stepping out into the field. Hence, the focus was more on conceptual and physical aspects of fidelity, and not psychological aspects of fidelity [10].

We felt a need to have an illustrated population from which a sampling frame can be identified and data can be collected. We considered the idea of a large illustration of a village (or settlement) with several households to make it amenable for sampling and data collection, and with each household holding within it a few data points. These data points would need to cover the types of variables—nominal, ordinal, discrete, and continuous. We wanted to keep the number of variables small and manageable, and hence, agreed to limit the number of variables to less than 10—corresponding to an equivalent number of survey questions.

### Step 2: tool design considerations

As the number of households (equivalent to houses for this project) in the village needed to be adequate to provide an opportunity to “sample” using various sampling techniques, we decided to keep the number as over 150 households. We decided to include three types of houses [*kachha, semi-pucca* and *pucca*] as defined by the Demographic and Health Survey in India (broadly indicative of socioeconomic status) [11]. We also wanted to ensure that the illustration fits into a regular sized classroom which may house up to 50 students—just the right size to make its presence felt while accommodating the number of houses needed.

### Step 3: tool design process

To help with the designing of various components, we collaborated with the communications team of the university who had expertize in illustrations and design—having supported other faculty members in designing pedagogical tools and games. After sharing the concept note, we held discussions with them to share the purpose, scope, and design expectations of the project.

**Fig. 1:**
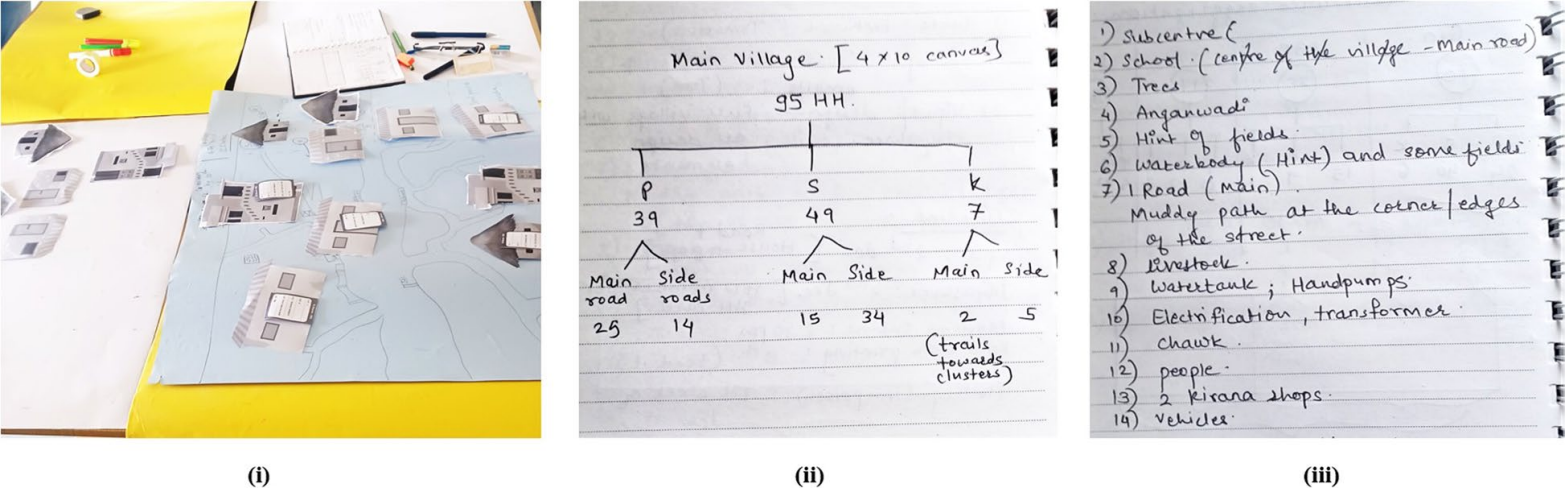
Glimpses of the designing process (i) discussing the size, structure, and layout of houses; (ii) discussing the frequency of various house types; and (iii) brainstorming about other structures to include in the village illustration

We envisaged the size of one house (which may determine the size of the overall village illustration) and possible ways of displaying the data against each household (see Fig. 1). We decided to display the data on “household cards” of the size of a visiting card; each of which could hold 5 data points (in response to five survey questions).

As more than one card can be made for each household and can be arranged in a stacked manner on the household; we decided to include socio-demographic variables and health variables displayed on two separate cards for each household. Based on this discussion, sample designs for three types of houses and for the data cards were made.

We finalized the size of the house and the card by keeping in mind the overall aesthetic appeal of the village illustration, easy readability, and ease of placing the cards on each house. After having shortlisted the house designs and card designs, the measurements revealed that we would be able to squeeze in between 150 to 160 households in an illustration of size 4.8 m (16 feet) by 1.2 m (4 feet). These dimensions would suit a classroom of length 6 m. We decided the breadth of the canvas should be limited to less than 1.5 m to allow physical access to each household (to be able to reach for the data cards with one’s hands) while standing beside the table on either side.

### Material

We finalized ‘canvas’ as the material for the village illustration due to the following reasons: (i) it is durable, (ii) the colours appear more attractive, and (iii) it can be rolled, carried, and easy to store. Similarly, the data cards for each household would be printed on 300 gsm art cards with matte lamination to ensure durability and clarity, with a unique color for each type of card to facilitate easier identification.

### Creating the dataset

We had several rounds of brainstorming sessions on creating the dataset, as the data would determine how many households of each type should be depicted in the village. The data on caste helped determine how the households would be clustered in the village as is largely seen in villages across India. In addition, we wanted to ensure that all types of variables are included across the two planned cards. We felt that the rates and prevalence need not exactly mirror those at the national level, but India’s average figures were used as a broad guideline to reverse engineer the data for this village, while keeping in mind adequate numbers under each category to make it useful. It resulted in a dataset with 10 variables for 155 households—incorporating broad considerations of how socioeconomic status relates to caste and health based on literature.

### Finalizing the tool

Using this dataset, we initiated the designing of the whole village—the laying out of the households keeping in mind their socioeconomic profiles, incorporating governmental services and infrastructure that would ordinarily be found in a village. Through an iterative process, we created the final layout based on the dataset.

## Description of the tool﻿

The *Surveypura* simulation pedagogical tool comes with (i) a large illustration of Surveypura village printed on a canvas, (ii) a set of household demographic cards (one card per household presenting the data for a few socio-demographic variables for that particular household), (iii) a set of household health cards (one card per household presenting the data on a few health variables for that particular household), (iv) a raw data file (so that the facilitator can become familiar with the data of all households to decide on how to use the tool and plan the assignments), and (v) the survey questionnaire used for this version of the dataset. The printing of all the material cost approximately USD 225 (as of early 2023). Details of the design and the logic behind it are provided below.

### The canvas (village illustration)

**Fig. 2:**
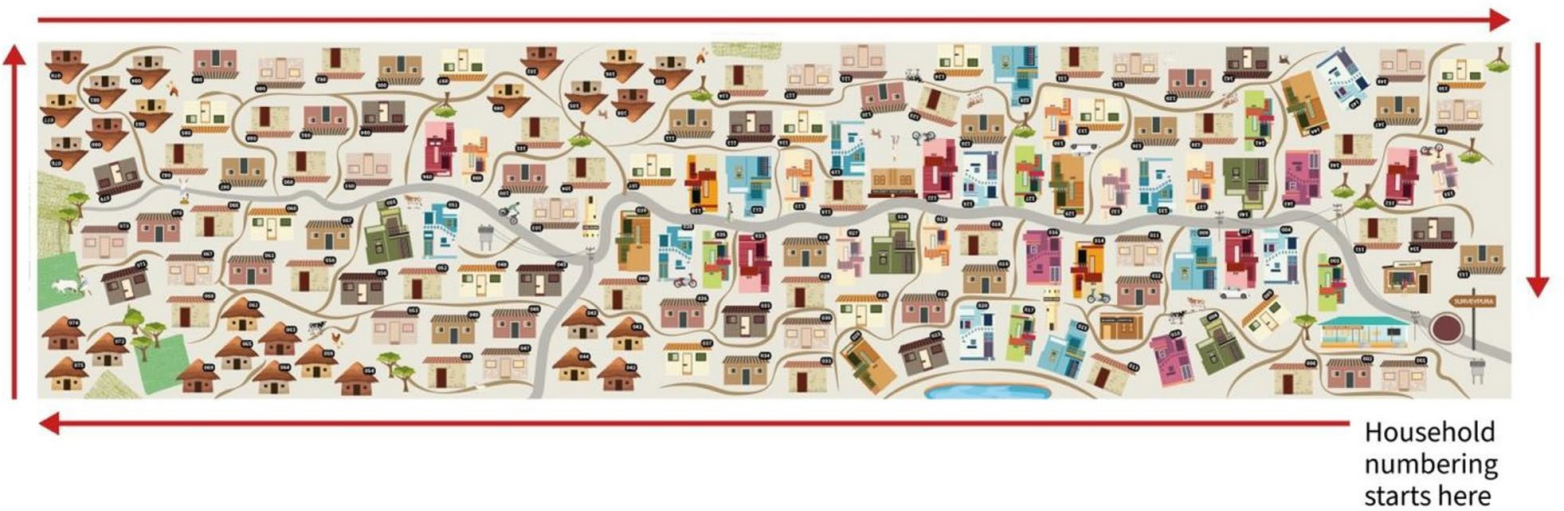
The illustration of Surveypura village. The direction of numbering of the households has also been indicated

The canvas is designed to give the students a feeling of being in a community setting. The canvas shows a rural setting in India, with paved and unpaved roads, houses, people going about their work, agricultural lands, waterbodies, trees, and livestock. It also has other features that may be seen in the average village such as a primary school, water tanks, shops, creche (*anganwadi*), and health sub-centre (see Fig. 2).

As the learner will be directly interacting with the sampled households on the canvas, the houses are positioned to face the learner. While Surveypura is a fictional village, the distribution of house types is reflective of inequalities and marginalization often seen in the Indian context. Also, while the village layout had pragmatic considerations, it is inspired by the villages that we have visited and from social maps we have encountered in our work.

Each household has been assigned a three-digit number [Household identification number or HHID], from 001 to 155, printed on the respective household. The numbering starts at the bottom right corner of the canvas, which is close to the brown *chowk* (crossroads or junction) and signpost that reads “Surveypura” (see Fig.﻿ 2).

### Household socio-demographic cards (demographic card)

**Fig. 3﻿:**
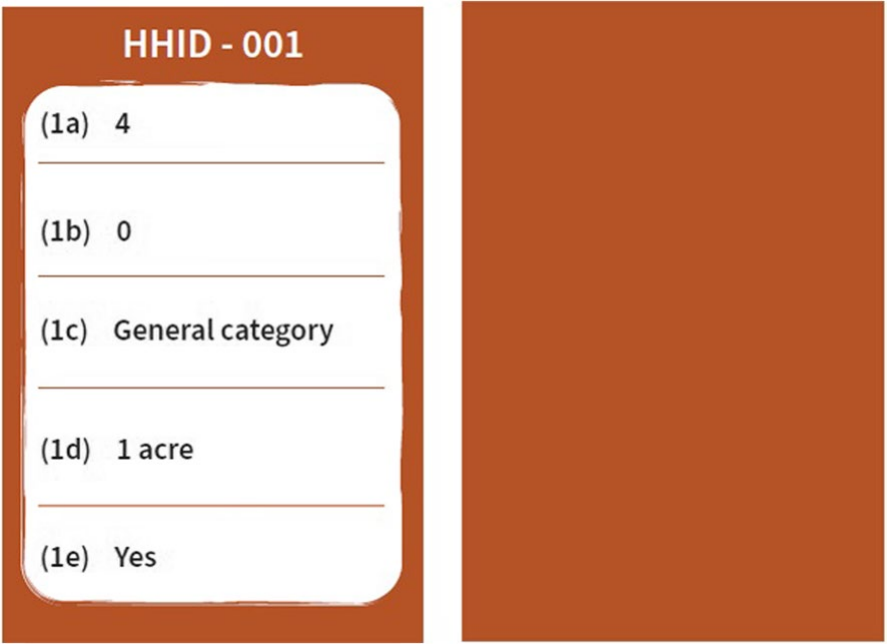
Sample household socio-demographic card (front and back respectively)

These cards are brown in colour, showing the HHID at the top which corresponds with a particular household on the canvas where each respective card is placed. On one side, the card provides the data of that household for five questions on sociodemographic characteristics (Questions 1a, 1b, 1c, 1d and 1e—please see the questionnaire in Additional file 1 and the sample card in Fig. 3 to understand better).

### Household health cards (health card)

**Fig. 4﻿:**
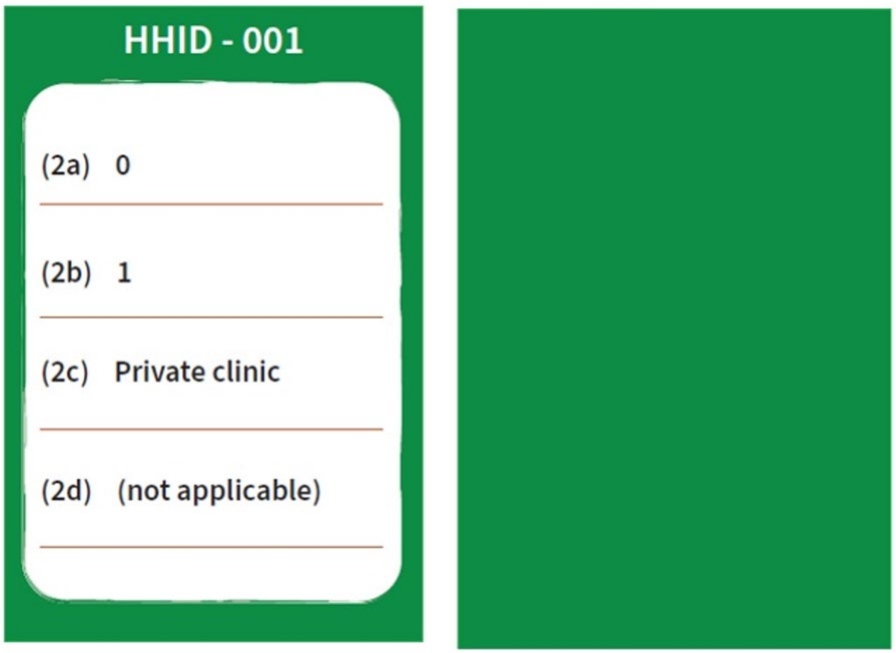
Sample household health card (front and back respectively)

Similar to the demographic cards, each of these cards corresponds to a particular household on the canvas. On one side, the card provides the data of that household for four questions related to the health of the members of the household (Questions 2a, 2b, 2c, and 2d—please see the questionnaire in Additional file 1 and the sample card in Fig. 4 to understand better).

### Survey questionnaire

The data on the demographic and health cards are answers to questions on a questionnaire (see Additional file 1) for the respective households. The questionnaire also includes two additional questions: household id, and type of house (ascertained by observing the illustration of that particular house). It is important to note that the questions have been deliberately kept few and simple. The idea is that this tool is simple enough to use and replicate, and can help students understand the basics. Details about how the tool can be used in teaching are provided in Additional file 1.

## *Surveypura* pilot and﻿ reflections

Prior to the start of the semester in January 2023, we introduced the tool to various course faculty at the university. Five faculty members teaching masters-level courses on quantitative methods for epidemiology and social research volunteered to use it in their classes.

### Pre-briefing﻿ and classroom exercises using *Surveypura:*

The course facilitators conducted a pre-briefing with the students. They were first taught concepts such as measurements, variables, and sampling techniques. They were then introduced to the tool (by taking a walk around the illustration and describing the features of the tool) and informed about how they would conduct the sampling and data collection in groups.

**Fig. 5:**
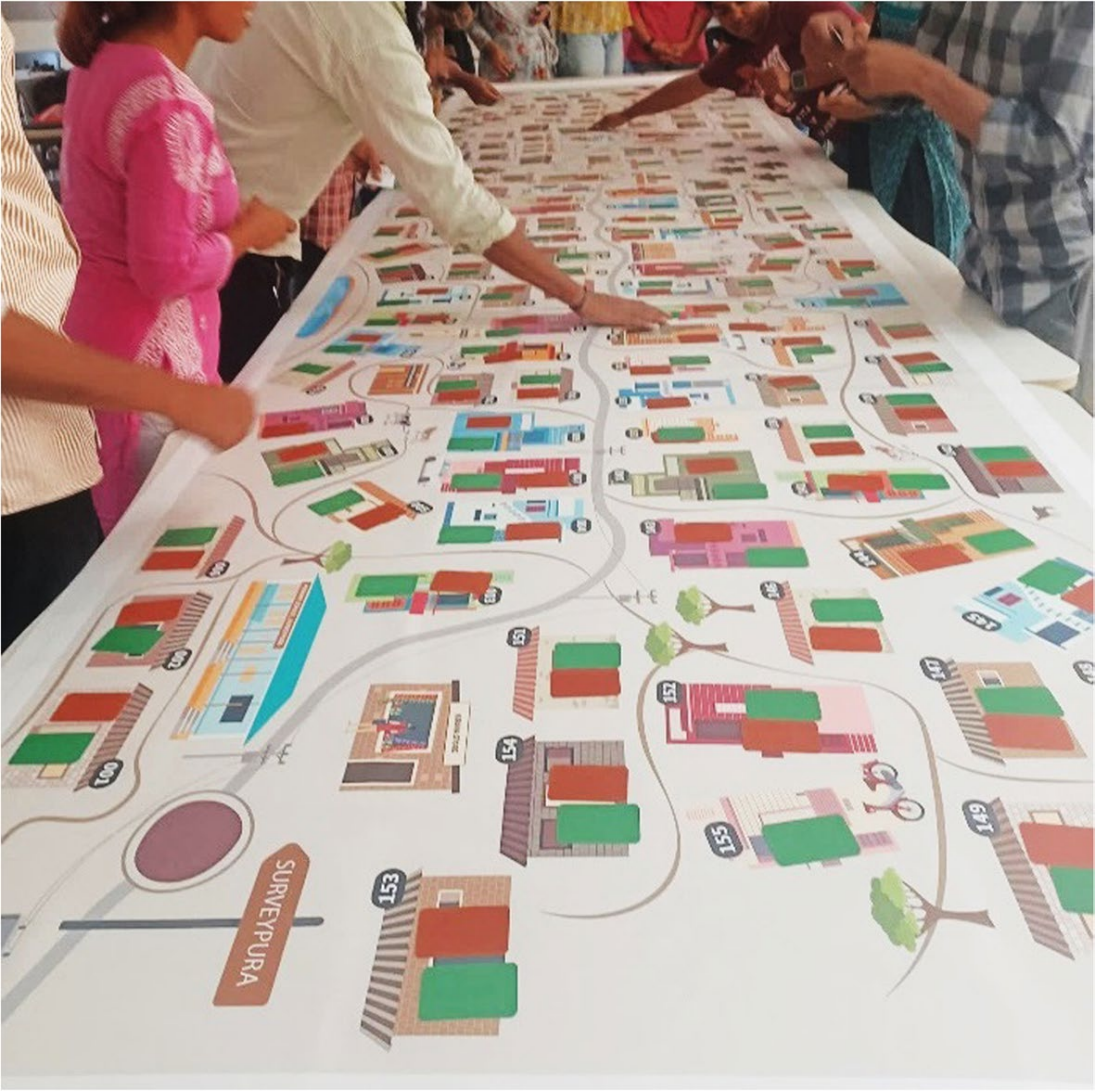
Students completing an exercise in quantitative methods that employed *Surveypura*

The students, in groups of 3 to 4, conducted probabilistic or non-probabilistic sampling of the households in Surveypura. This was followed by the collection of data on select variables using the demographic and health cards of the sampled households (using, for instance, online survey tools), which was later used to further discuss concepts of sampling, data analysis, inference, and reporting. The facilitators (alongside us) were available to clarify and questions during the exercise (see Fig. 5).

### Insights from observation and de-briefing

With the objective of understanding the application and usefulness of the tool in teaching quantitative methods to students pursuing higher education, we (i) observed the lectures (see Fig. 5) whenever the tool was used (five lectures of two hours each, with class size ranging from 13 to 40 students), and (ii) conducted debriefing with two-course facilitators and five students (with prior informed verbal consent) a few weeks after the tool was used in their class given their availability.

Regarding our method of observation during the class exercises, we made detailed notes on the pre-briefing, the application of *Surveypura*, students’ engagement with the tool, and any reported or observed concerns related to the content and design of the tool. The two authors then compared and compiled their notes.

The debriefing covered reflection-on-action [12], alongside inputs on the design of the tool and the pedagogy used. The topics covered were: preparation undertaken for using the tool, ease of application, and reflections on (i) design aspects of the village and cards, (ii) the dataset and variables, (iii) class engagement and learning, and (iv) relevance for teaching and learning of quantitative methods. For each of these topics, both the perceived benefits and constraints were explored with the respondents.

The findings from our observation notes and debriefing interactions are presented below:

### Engaged and non-intimidating learning experience using *Surveypura*

Facilitators’ efforts in setting up the context prior to using *Surveypura* was found to be important. Providing an idea about the tool and access to questionnaire along with a discussion on the theoretical concepts prior to using the tool helped students appreciate the experiential learning process better. Most of the student participants expressed that consistent use of *Surveypura* would ensure better retention of learnings of basic quantitative methods. Consistent use would also help them better appreciate how different steps in a cross-sectional study are connected to each other in real life.

It was observed that the use of the tool spread in the center of the classroom nudged a collaborative and fun ‘learning by doing’ experience among students, which was deemed important by students who struggle with quantitative skills.

One facilitator shared,

“...it helps take away their fear for numbers which many times keeps them disengaged in class. This is especially important for students who come from disadvantaged backgrounds who may not always have enough resources to learn good mathematics.”

The finding on the creation of engaging environment in the classroom was corroborated as one student shared,

“We are usually sitting in the class, but while using Surveypura, we were standing, we were finding things, so we needed to be alert… so the process was more involved than usual. In my previous course, I had learnt sampling in a theoretical manner. But this time using the tool, it was much more enriching and gave me more confidence to know how to do it.”

Some students reported clearer understanding of previously introduced concepts following the session using *Surveypura.* Most students and facilitators felt that Surveypura has the potential to provide rich learning experience as it develops the skills of ‘doing’ various steps in the quantitative methods, such as sampling, entering the data on spreadsheets, calculating proportions, along with the visual appeal it provides. As the tool is kept in the middle of the classroom on a set of arranged tables surrounded by the students facing each other, it facilitates interaction among students. One facilitator mentioned,

“Usually, slides or posters are on the wall - on the side... but this was in the centre, which also creates a lot of curiosity, excitement. Then students are not stressed out… If learning is intimidating, then learning is tiring. Surveypura helps take away that intimidation.”

While students acknowledged that the human interaction element of an on-field survey cannot be brought into this tool and that each household’s data was readily available in *Surveypura* (in terms of data cards), the tool provided an experience of planning and conducting a survey while still being inside a classroom.

### Pedagogy planning for effective usage of *Surveypura*

Our findings underscore a strong need for planning the pedagogy in advance for using *Surveypura* in class. This includes getting acquainted with the features of the tool, creating the exercises, and introducing the tool to the students. While the facilitators appreciated the detailed manual that was prepared, they felt it would help to have an audiovisual resource for first-time users of the tool. One facilitator shared that session planning took about 8–10 h as the tool was used for the first time. They mentioned,

“A lot of thought process went into deciding how to demonstrate each sampling method (such as)…what sample size is needed to demonstrate this… Also, we needed a way to show which households had been sampled – so, I used poker chips to mark the selected households on the canvas. This can also give a broad visual idea of how it may look if a particular sampling method is used. I also took a few variables from the questionnaire and created the sample estimates against that of the population. I also created the forms on Kobo Collect (software).”

One facilitator felt that this initial effort would reduce the preparation time for the next use of the tool. Finally, there was an observation of the lack of individual-level data in the existing data, which limits the ability to calculate some measures. We were aware of this limitation, and feel that this can be overcome by creating another set of cards for individuals in the household.

### Appropriate class-size for *Surveypura*

While it was found that *Surveypura* contributed to creating an engaging environment to learn quantitative methods, the engagement seemed to be influenced by the class size. The range of class sizes varied from 13 to 40. The facilitator with 13 students felt that they were able to create a relaxed learning environment, and students engaged more actively. However, students who were a part of a large class felt that it was crowded to conduct the exercise using *Surveypura* with many people around. The opinions of our respondents corroborated our observations and reflections that a class size of around 20 would be appropriate for better engagement and use of the canvas.

We recognize that having conducted the observation and debriefing of our tool ourselves may have influenced the findings (i.e., interviewer bias). However, we note that the facilitators did not hesitate to discuss the constraints of the tool. Also, the student respondents graduated just before the debriefing, and hence, we believe there was little reason to deceive us with unwarranted flattery.

### Flexibility for adaptation to other contexts

Learning facilitators from other contexts can quite easily modify the design elements of the village illustration and the data on the cards to make them relevant to their contexts. For instance, instead of depicting an Indian village, an urban poor neighborhood in a city or a village in the African region can be illustrated; and similarly, the variable and the data on the caste category of the household could be replaced with another contextually relevant variable such as race. There is also a scope for designing new card sets presenting data on different domains such as livelihood, migration etc. as required.

## Conclusion

Having recognized the challenges faced by several students in learning about quantitative methods, we developed a simulation pedagogical tool *Surveypura*. It was designed for easy use and cost-effective reproducibility. The initial experiences of employing the tool revealed that the facilitators and students found it useful, and exciting, especially for topics such as sampling and data collection. Debriefing with participants revealed that the tool boosts a collaborative learning experience, which is deemed to be a valuable competence in different health professions [13]. Various aspects of the tool including its design, scope, and aesthetics were appreciated by the participants for contributing to an experiential learning environment. While the role of the facilitator in reducing the fear is undeniable, tools such as *Surveypura* can contribute to creating a more welcoming learning environment, especially for those who might come with a fear of numbers.

While we were able to observe some of these strengths of the tool, the limitations in terms of the class-size it can satisfactorily accommodate, the time needed for facilitator preparation, and current limitations in the individual-level data were also recognized. We have discussed ways in which these limitations could be addressed. Overall, having observed its application in class, and having heard from several people who were exposed to the tool, we feel optimistic about the usefulness of *Surveypura*, not just in India, but also outside it, after minor adaptations.

### Supplementary Information

**Additional file 1.** (1) Questionnaire that was used for this version of *Surveypura* (formatted for use); (2) Suggested steps to use Surveypura in the classroom; (3) Incomplete list of topics that can be discussed using *Surveypura*.

## Data Availability

The data from the debriefing interactions is available upon request. The soft copy of the simulation pedagogical tool itself will be made available free of cost on the Azim Premji University website along with the manual.
