# Supplementary material for: Reflections on the design and application of ‘Surveypura’: a simulation-based pedagogical tool for quantitative research methods in public health and social sciences
Source: Adv Simul (Lond). 2024 Jan 7;9:2. doi: 10.1186/s41077-023-00275-y (PMC10773104; doi:10.1186/s41077-023-00275-y)
Supplement: Supplementary file 1 — Additional file 1. (1) Questionnaire that was used for this version of Surveypura (formatted for use); (2) Suggested steps to use Surveypura in the classroom; (3) Incomplete list of topics that can be discussed using Surveypura. [file 41077_2023_275_MOESM1_ESM.docx]

**Additional file 1**

1. Questionnaire that was used for this version of *Surveypura* (formatted for use)

Please read the below questionnaire carefully. It has three sections-

Observation section: Prior to noting down the responses to the answers 1a-1e and 2a to 2d, you need to write down the responses to questions 0a and 0b, which are to be done by carefully observing the Surveypura canvas.

Section 1: Sociodemographic questionnaire [responses for this can be found on the brown cards]

Section 2: Health questionnaire [responses for this can be found on the green cards].

Please pay attention to the column ‘Go to’ in sections 1 and 2 which indicates any skips in the questionnaire.

| **Observation section** | | | |
| --- | --- | --- | --- |
| **0a** | **HHID** | [Observe the ID written on the house and enter] |  |
| **0b** | **What type of house is this?** | [observe the characteristics of the house and choose from the below options]  Kachha house…………………… 1  Semi-pucca house……………… 2  Pucca house…………………..… 3 |  |
| **Section 1: Sociodemographic questionnaire** | | | |
| Question number | Question | Response | Go to |
| **1a** | **What are the total number of persons living in the household?** | \|  \|  \| \| --- \| --- \|   The total number of persons living in this household…  [enter the number in the box or choose the below option as per the response on the card]    Respondent not at home. Neighbors say they are migrated……………………….999 | **Question 1b**  END THE FORM |
| **1b** | **How many household members are under the age of 5 years?** | … The number of children under age 5 years [enter in the box]   \|  \|  \| \| --- \| --- \| |  |
| **1c** | **What is the caste category of the head of the household?** | [choose from the below options]  General caste……………... 1  Scheduled caste………… 2  Scheduled tribe……….… 3  Other backward classes…. 4 |  |
| **1d** | **What is the total land owned by the household? (in acres)** | … Total land owned in acres [enter in the box]   \|  \|  \| \| --- \| --- \| |  |
| **1e** | **Does the household have a toilet** | [choose from the below options]  Yes……… 1  No…….… 2 |  |
| **Section 2: Health questionnaire** | | | |
| **2a** | **Since past one year [from the date of the interview] how many members of the household got diagnosed with Tuberculosis?** | Number of members in the household got diagnosed with Tb in past 1 year [enter in the box]   \|  \|  \| \| --- \| --- \| |  |
|  |  |  |  |
| **2b** | **How many members in your household have been diagnosed with diabetes mellites?** | Number of members in the household diagnosed with diabetes mellites [enter in the box]   \|  \|  \| \| --- \| --- \| |  |
| **2c** | **When members of your household get sick, where do they generally go for treatment?** | [choose from the below options]  Primary health centre………… 1  Private clinic…………………... 2  Traditional healer…………..… 3 |  |
| Check Section 1,   \| 0 \| 0 \| \| --- \| --- \|   IF the response to Question1b is THEN END THE FORM; else continue | | | |
| **2d** | **Total number of children under the age of 5 years identified as undernourished? (Based on Anganwadi data)** | The total number of children <5 years identified as undernourished  [enter]   \|  \|  \| \| --- \| --- \| |  |
| **END THE FORM** | | | |

1. Suggested steps to use *Surveypura* in the classroom

There are several ways in which *Surveypura* can be used in teaching quantitative research methods – we have discussed a few such ideas later in the manual. Below, we describe what we anticipate as the primary use of this version of *Surveypura*.

### *2.1. Requirements*

Below are the requirements to use *Surveypura:*

- a room that can accommodate the 16ft*4ft canvas (this could also be done outdoors in a shaded area too, but wind can be a problem for placing the cards on the houses)
- the canvas with the illustration of Surveypura (measuring 16ft*4ft)
- adequate number of tables arranged together to display the canvas
- the card sets (one can decide to use only the sociodemographic cards, or both sets of cards)
- the questionnaire – in the electronic or paper format (this can also be done as part of the class activity to help learners design an electronic questionnaire)

### *2.2. Pre-exercise preparation*

The primary purpose of Surveypura is to help learners understand the concepts and some practical aspects of a cross-sectional study – specifically, in planning, conducting, interpreting and reporting a survey. Learners are introduced to the cross-sectional study design. This may include aspects such as use of the design, measures, sampling strategies, challenges etc. The facilitator should become fully familiar with the canvas, the cards, the dataset, and the questionnaire so that the exercise can be planned well.

The objective of the cross-sectional study based on the included dataset is to understand the socio-demographic and health status of Surveypura. The questionnaire is shared with the learners to stimulate the discussion on framing of questions and types of variables. The learners, either individually or in small groups, can then prepare the electronic version of the questionnaire or print adequate number of paper questionnaires. Following that, students “visit” each assigned household on Surveypura and can enter the data by referring to the data cards available on the assigned households. There is the option of assigning a random sample of households to some students, and biased samples to some. Following that, the learners can create an analysis plan for when the data is collected.

An important point that should not be forgotten is for the facilitator to have a detailed discussion on the ethics of quantitative data collection. It should not be assumed by the students or the facilitator that one can walk to households or individuals and just expect data to the given. This aspect could be dealt with in many ways, for instance, discussing about the ethical dimensions of the consent and the included questions and variables, and also by encouraging students to prepare a consent form for the survey.

### *2.3. During the exercise*

In the room where the exercise will be conducted, the tables can be arranged juxtaposed with each other so that we have a surface of 16ft*4ft upon which the canvas can be displayed. The canvas is then displayed on the tables. The learning facilitator can then place the demographic or both cards (as needed) on top of the door of each respective household (card with HHID-001 on the door of house 001 and so on).

Now that the cards are placed against all households, the students can take some time to familiarise themselves with the illustration. They can be asked to observe the village and report their observations. The learners can then be informed about how the houses have been numbered, so that they can find their “respondents” [sampled households] more easily. It is important to tell the learners that they should replace each card in the same spot once the data has been collected from that particular household – so that other learners can visit that household if need be.

Learners are then given time to collect the data, by “visiting” each sampled household, checking the respective cards and making the entries in their smartphones, tablets, or paper questionnaires. It is suggested that the data entry be done using an electronic format to reduce errors and effort. The facilitator can be around to answer any doubts. Students should be asked to note down any doubts that arise, difficulties faced, or observations made. Students can also be told not to make their entries too quickly as it can lead to errors.

### *2.4. After the data collection*

Learners will download the spreadsheet with the collected data and observe it. After familiarising themselves with the data, they will analyse the data under guidance of the facilitators, using their analysis plan. Facilitators can then discuss the results with the learners and use the opportunity to further consolidate their understanding of various concepts related to quantitative research methods. Some ideas for possible discussion topics have been listed in the next section. Students can then be asked to prepare a report based on their results and inferences.

1. Incomplete list of topics that can be discussed using *Surveypura*

This list is not exhaustive, and the topics that can be discussed using this tool is dependent on the creativity of the learning facilitator. Given below are some ideas:

- 1. **Household listing:**
  - How is household listing done?
  - Why do we need to do household listing?
  1. **Sampling:**
  - What is sampling?
  - How is it done?
  1. **Understanding questionnaires**
  - open/closed questions, skips, range of possible answers etc.
  - different types of variables
  1. **Using observation for data entry:**
  - The variable “type of household” is based on the learner’s observation.
  1. **Linking the entered data on the spreadsheet with the act of data collection**
  - How each row corresponds with a particular respondent/household
  1. **Missing data and data entry errors**
  - e.g. Whether missing data is valid skip or a data entry error?;
  - If people are not at home do we revisit that house or do we collect the information from their neighbours?
  1. **Calculating quantitative measures**
  - Proportion – what is it? How to calculate it? [for example, proportion of households owning toilets]
  - Mean – what is it? How to calculate it? [for example, mean household land ownership]
  - Prevalence – what is it? How to calculate it? [for example, prevalence of diabetes]
  - Incidence – what is it? How to calculate it? [for example, tuberculosis incidence]
  - Ratio – what is it? How to calculate it?
  - Odds – what is it? How to calculate it?
  1. **Analysis and interpretation**
  - Disaggregating and comparing the data – what does it tell us? What are the limitations of the data and the methods?
  - Generalizability**:** is this data representative of the population? What are the alternative explanations for the findings?
  1. In addition, there is scope for discussing about access to village amenities, social mapping, geographic and livelihood aspects etc.
